# The Transcriptional and Protein Profile From Human Infected Neuroprogenitor Cells Is Strongly Correlated to Zika Virus Microcephaly Cytokines Phenotype Evidencing a Persistent Inflammation in the CNS

**DOI:** 10.3389/fimmu.2019.01928

**Published:** 2019-08-16

**Authors:** Morganna C. Lima, Leila R. de Mendonça, Antonio M. Rezende, Raquel M. Carrera, Conceição E. Aníbal-Silva, Matthew Demers, Leonardo D'Aiuto, Joel Wood, Kodavali V. Chowdari, Michael Griffiths, Antonio R. Lucena-Araujo, Manoel Barral-Netto, Elisa A. N. Azevedo, Renan W. Alves, Pablo C. S. Farias, Ernesto T. A. Marques, Priscila M. S. Castanha, Claire L. Donald, Alain Kohl, Vishwajit L. Nimgaonkar, Rafael F. O. Franca

**Affiliations:** ^1^Oswaldo Cruz Foundation/Fiocruz, Institute Aggeu Magalhães, Recife, Brazil; ^2^Institute of Infection and Global Health, University of Liverpool, Liverpool, United Kingdom; ^3^Department of Psychiatry, University of Pittsburgh School of Medicine, Pittsburgh, PA, United States; ^4^Federal University of Pernambuco/UFPE, Recife, Brazil; ^5^Oswaldo Cruz Foundation/Fiocruz, Institute Gonçalo Moniz, Salvador, Brazil; ^6^Center for Vaccine Research, University of Pittsburgh, Pittsburgh, PA, United States; ^7^MRC-University of Glasgow Centre for Virus Research, Glasgow, United Kingdom; ^8^Department of Human Genetics, Graduate School of Public Health, University of Pittsburgh, Pittsburgh, PA, United States

**Keywords:** Zika virus, central nervous system, inflammation, type-I interferon, interferonopathy, microcephaly, Zika congenital syndrome and cytokines

## Abstract

Zika virus (ZIKV) infection during pregnancy is associated with microcephaly, a congenital malformation resulting from neuroinflammation and direct effects of virus replication on the developing central nervous system (CNS). However, the exact changes in the affected CNS remain unknown. Here, we show by transcriptome analysis (at 48 h post-infection) and multiplex immune profiling that human induced-neuroprogenitor stem cells (hiNPCs) respond to ZIKV infection with a strong induction of type-I interferons (IFNs) and several type-I IFNs stimulated genes (ISGs), notably cytokines and the pro-apoptotic chemokines CXCL9 and CXCL10. By comparing the inflammatory profile induced by a ZIKV Brazilian strain with an ancestral strain isolated from Cambodia in 2010, we observed that the response magnitude differs among them. Compared to ZIKV/Cambodia, the experimental infection of hiNPCs with ZIKV/Brazil resulted in a diminished induction of ISGs and lower induction of several cytokines (IFN-α, IL-1α/β, IL-6, IL-8, and IL-15), consequently favoring virus replication. From ZIKV-confirmed infant microcephaly cases, we detected a similar profile characterized by the presence of IFN-α, CXCL10, and CXCL9 in cerebrospinal fluid (CSF) samples collected after birth, evidencing a sustained CNS inflammation. Altogether, our data suggest that the CNS may be directly affected due to an unbalanced and chronic local inflammatory response, elicited by ZIKV infection, which contributes to damage to the fetal brain.

## Introduction

Congenital Zika Syndrome (CZS) comprise a wide spectrum of birth defects and symptoms observed in infants who have been exposed to Zika virus (ZIKV) during embryonic development (1). Although other minor abnormalities have been documented, the most dramatic symptoms are microcephaly, severe microcephaly, arthrogryposis, and ocular damage (2). Microcephaly is characterized as a developmental brain malformation that results in a cranial circumference <2 standard deviations (2-SD) below the average for the same sex and gestational age, on the same way, severe microcephaly is classified based on a cranial circumference <3-SD below the average (3, 4). Microcephaly associated with ZIKV infection in pregnancy was first reported in Brazil in 2015 during a large outbreak of this virus (5). Initial associations between ZIKV infection and microcephaly development were based on the identification of viral RNA in fetal amniotic fluid (6) and in the brain tissue of fetuses and infants diagnosed with microcephaly (7, 8). Later, several reports from independent groups confirmed the association between ZIKV infection during pregnancy and congenital abnormalities (9).

Diagnosis of CZS is complex and involves several different steps, including taking head circumference measurements, neurological evaluation, radiologic brain imaging and ophthalmologic assessment. ZIKV exposure confirmation can be accomplished using molecular and serologic standard methods. However, since ZIKV is present in body fluids with different shedding kinetics, confirmation of virus infection remains challenging (10). In neonates presenting with signs of microcephaly, ZIKV infection can be confirmed by virus detection (i.e., RT-PCR) or by the presence of ZIKV-specific IgM in cerebrospinal fluid (CSF) or serum (4, 11). Importantly, the presence of anti-ZIKV IgM in the CSF of neonates with suspected CZS is a strong indicator of a recent congenital infection (12). Although significant improvements have been reached in terms of diagnosis, the exact pathogenic mechanisms related to ZIKV-induced microcephaly and others malformations remain largely unknown. Emerging evidence demonstrated that ZIKV infection impairs brain development by arresting cellular neurogenesis, leading to the deregulation of cell-cycle progression and apoptosis (13–16). Moreover, previous work has also assessed the birth defects resulting from ZIKV exposure in different pregnant mouse models (17–20). While most studies are focused on elucidating the specific cellular mechanisms of ZIKV pathogenesis through *in vitro* or *in vivo* models, the pathogenesis among naturally infected human subjects and its biological implications have not been investigated to the same extent.

In humans, the central nervous system (CNS) becomes to be established from 22 days onwards. Initially, the embryonic brain is entirely composed of highly proliferative neuronal progenitor cells (NPCs). Thus, at this stage, pathogenic processes induced by the unbalanced production and local secretion of immunoregulatory molecules may lead to reduced brain size, and consequently microcephaly (21). Therefore, investigations into ZIKV triggered immune responses, especially in the CNS, may contribute to a better understanding of the disease mechanisms since specific immune mediators may play a major role in the pathogenesis of microcephaly. The finding that ZIKV infection leads to apoptosis in different neuronal models could also partially explain the cellular destruction observed in the radiological examination of neonates with CZS (22). On the other hand, the paracrine effects of cytokines and chemokines directly secreted in the CNS by the infected cells are still unknown. More recently, Tappe et al. (23) assessed cytokine kinetics in the serum of ZIKV infected patients. Despite the relatively small number of patients analyzed, the authors were able to compare samples from the acute and convalescent-phase, observing elevated levels of the chemokines CCL3, CCL4, CCL5 and CXCL10 during the recovery phase. During the acute phase, the authors observed a mixed cytokine pattern, with the increase of cytokines profiles associated with Th1, Th2, Th17, and Th9 CD4^+^ T cell responses (23). In addition, it was reported that there were increased levels of CXCL10 and CCL2, IL-6, IL-8, VEGF, and G-CSF in the amniotic fluid of ZIKV-positive pregnant women with neonatal microcephaly (24). Furthermore, the overexpression of CXCL10 was recently identified as a potential serum biomarker of acute ZIKV infection (25). Taken together, these findings may suggest that ZIKV infection results in a specific pro-inflammatory profile. However, no reports from microcephaly cases were available so far, and it remains to be elucidated whether ZIKV-induced microcephaly could be associated with an organ-specific inflammation.

Here, we analyzed the transcriptional changes induced by ZIKV infection in human induced pluripotent neuroprogenitor stem cells (hiNPCs). We used this model to compare the pathogenesis of a contemporary South American strain (isolated in Brazil in 2015 at the peak of microcephaly cases) vs. an ancestral Asian strain isolated in Cambodia in 2010. Transcriptional data were further confirmed at protein expression levels and by assessing the levels of cytokines and chemokines in the CNS of confirmed ZIKV-induced microcephaly cases and validated through a second *in vitro* infection assay in human neuroblastoma cells. Our findings suggest an important role of type-I interferon (IFN) response and chemokines CXCL10 and CXCL9 in the pathogenesis of microcephaly, which may represent a still unaddressed target with the potential to interrupt the destructive CNS inflammation induced by ZIKV infection.

## Methods

### hiNPCs Culture and Infection

Human induced neuroprogenitor cells (hiNPCs) derived from human induced pluripotent stem cells (hiPSCs), line 73-56010-02 sub-clone F, were grown on matrigel-coated, cell culture treated plates. Briefly, hiPSCs, were cultured with neuronal precursor selection medium, followed by neuronal precursor expansion medium (Thermo Fisher Scientific, Munich, Germany) containing fibroblast growth factor 2 (R&D Systems, Minnesota, USA) for the generation of neural stem cells. After 5–7 days in culture, neural rosettes were identified, manually dissected and plated into low-attachment plates where embryoid body-like structures—denoted as neurospheres—emerged. Following the plating of neurospheres into the matrigel-coated plates, hiNPCs were collected manually for monolayer culture. hiNPCs were then cultured in neurobasal medium containing B27 (Thermo Fisher Scientific, Munich, Germany) supplement and brain-derived neurotrophic factor 10 ng/mL (R&D Systems, Minnesota, USA) for neuronal differentiation. hiNPSCs cultured cells, passage 5, were grown until 85% confluent in 12-well plates and further infected at (multiplicity of infection) MOI 1 with a ZIKV Brazilian strain (*ZIKV/H.sapiens/Brazil/PE243/2015, GenBank: KX197192.1*), previously described (26) or a ZIKV Cambodian strain (Zika virus isolate Cambodia FSS13025/2010, GenBank: JN860885.1) prepared in Vero E6 cells, previously established in our laboratory. At 1-h post infection, the inoculation medium was removed and replaced with culture medium. At 48 h post infection, the cells were harvested and RNA was extracted for RNA-sequencing (RNA-Seq) and transcriptomic analysis.

### RNA-Seq and Data Analysis

RNA-Seq was performed at the Genomics Research Core, University of Pittsburgh, USA. Before sequencing, RNA quality was checked using the Agilent High Sensitivity RNA ScreenTape System (Agilent Technologies, Santa Clara, USA). Whole-transcriptome sequencing was performed using the TruSeq Stranded Total RNA kit (Illumina Inc., San Diego, CA, USA) with an average of 49.1 million reads per library. Each condition (infected and control samples) was sequenced in triplicate. The quality of sequenced reads was assessed by FastQC tool. Reads from each library were mapped against the human genome assembly GRCh38 with the annotation version 91 downloaded from the Ensembl database, applying the STAR aligner version 2.5.3. Later, R package DESeq2 was used to perform differential expression analysis (DEA). During this step, the biological replicates from infected and control samples were compared, but only genes presenting at least ten reads for all three biological replicates in at least one condition were considered for DEA. Genes with an absolute value of log_2_ fold change equal to or >1 and with a *p*-value, correct by the FDR approach, of <0.05 were selected for functional analysis using the STRINGdb R package (http://www.string-db.org) and *KEGG REST* for pathway-based data integration (https://www.kegg.jp/kegg/rest/). The former was used to assign Gene Ontology (GO) (http://geneontology.org/) and Kyoto Encyclopedia of Genes and Genomes (KEGG) pathway terms to differentially expressed genes (DEGs). In addition, the *Biological Process* ontology of GO was employed to perform a functional enrichment analysis for those genes. KEGG pathways were grouped into four categories (adaptive immune response, cytokine and chemokine signaling, interferon response, and cell death and growth) to visualize protein networks present in the STRING database. The protein networks built from the lists of DEGs were analyzed using Cytoscape software, version 3.6.0 (https://cytoscape.org/). Heatmaps were built using the gene counts normalized by the library size factors using the function heatmap of R environment. The mean of gene expression fold change from infected cell samples were plotted vs. the expression found in non-infected ones (Control) and visualized with ViaComplex software (27) (http://lief.if.ufrgs.br/pub/biosoftwares/viacomplex). For this analysis, we selected only the list of modulated genes identified in Figure 2A.

### Infection of Neuroblastoma Cells and Cytokine Analysis

Human undifferentiated neuroblastoma cells (cell line SH-SY5Y-ATCC® CRL-2266™) was cultured in 1:1 MEM and Ham's F12 Nutrient Mixture, supplemented with 1 mM sodium pyruvate, 2 mmol/L L-Glutamine, non-essential amino acids, 100 U/mL penicillin, 100 μg/mL streptomycin (Thermo Fisher Scientific) and 10% (v/v) fetal bovine serum in a humidified incubator at 37°C with 5% CO_2_. Cells were infected with ZIKV/Brazil (*ZIKV/H.sapiens/Brazil/PE243/2015, GenBank: KX197192.1*) and analyzed at different days post-infection (dpi), as described on figure legends. Briefly, cells were harvested at 2 dpi and stained with anti-flavivirus envelope (E) protein primary antibody (4G2) and goat anti-mouse IgG secondary antibody conjugated with FITC (Sigma-Aldrich, St. Louis MO), and analyzed by flow cytometry (FACS). Supernatants were harvested and processed for viral RNA extraction and qRT-PCR assays in several time points post-infection. In addition, supernatants were also harvested at 3 dpi and soluble cytokines and chemokines were quantified by the kit Cytometric Bead Array (CBA) Human Inflammatory Cytokine and Human Chemokine Kit (BD Biosciences, San Diego, CA, USA), following the manufacturer's instructions.

### Patients

Cases of microcephaly and other birth defects enclosing the CZS cases notified from 2015 to 2017 were investigated following a previously established protocol from the Brazilian Ministry of Health as follows: neonates with suspected microcephaly were investigated by measuring their head circumference. Those with a circumference of at least 2 SD below the mean for the same sex and gestational age on the Fenton growth chart were diagnosed with microcephaly. Severe microcephaly was diagnosed in those infants who presented with a head circumference smaller than 3 SD. Brain imaging was performed whenever possible. Control subjects were live neonates with suspected microcephaly at birth who had a CSF sample collected to perform ZIKV diagnosis, which were later classified as healthy through transfontanellar ultrasonography of the brain and which presented no other major birth defects. Exclusion criteria were anencephaly, encephalocele, and the confirmation of the phenotype of a well-defined congenital syndrome. Congenital infections, other than ZIKV, were assessed in infants and mothers from a blood sample collected right after birth by the STORCH laboratory-testing panel (congenital infections encompassing syphilis, toxoplasmosi*s*, rubella, cytomegalovirus and herpes simplex virus). CSF samples from all neonate CZS suspected cases were collected by lumbar puncture, samples collected at a maximum of 4 weeks after birth were included in this study. For ZIKV infection confirmation, CSF samples were forwarded to the Arbovirus Reference Laboratory at the Oswaldo Cruz Foundation, Fiocruz/Recife, Brazil. ZIKV infection was diagnosed by detection of IgM antibodies or by ZIKV RNA presence determined by Real Time RT-PCR (rRT-PCR). ZIKV exposure was confirmed based on a positive laboratory result from CSF and/or serum.

### Multiplex Immunoassays

Individual cytokines in all human neonate CSF samples were assayed in a Luminex Platform employing a commercially available kit: Cytokine Human Magnetic 25-Plex Panel (Thermo Fisher Scientific, Munich, Germany) following the manufacturer's instructions. hiNPC-derived infected supernatants were virus-inactivated by homogenizing samples in NP-40 at 0.2% and analyzed by the Human Cytokine 42-Plex Discovery Assay (Eve Technologies, Calgary, Canada). A complete list of the analyzed cytokines and chemokines and its detection limits are described in Supplementary Material (Tables S2, S3).

### Ethics Statement

Ethics protocol and procedures have been reviewed and approved by Institutional Ethics and Research Committee of the Institute Aggeu Magalhaes, Oswaldo Cruz Foundation–Fiocruz, Brazil (CAAE: 73669417.7.0000.5190). The hiNPC-based studies were approved by the University of Pittsburgh internal IRB and IBC.

### ZIKV ELISA IgM

ZIKV IgM antibodies were detected by MAC-ELISA (Capture Enzyme-Linked Immunosorbent Assay), employing an in-house protocol. Briefly, ZIKV antigen was prepared from ZIKV-infected Vero E6 cells. ELISA plates were sensitized with Goat anti-human-IgM (KPL/Sera Care, Milford, USA), before samples were added (1/400 dilution). Reactions were performed employing a detection antibody (MAB 6B6C-1/HRP), kindly provided by CDC (Centers for Disease Control and Prevention, Atlanta, USA). Serum and CSF samples were considered positive when the optical density exceeded 3.0 times that of the negative control.

### Virus Detection–Real-Time RT-PCR and Plaque Assays

Viral RNA was extracted manually from human serum and CSF samples using a QIAamp Viral RNA kit (Qiagen, Hilden, Germany), following the manufacturer's instructions. ZIKV Real Time RT-PCR (rRT-PCR) reactions were performed from purified RNA serum samples employing primers and probes as described by Lanciotti et al. (28). Briefly, reactions were performed in duplicate in a final volume of 20 μl employing the kit GoTaq® Probe 1-Step RT-qPCR (Promega Corporation, Madison, USA), following manufacturer instructions. Cycling was performed using the QuantStudio 5 Real-Time system (Thermo Fisher Scientific) and samples with a Ct value <38 in duplicate wells were considered to be positive for ZIKV. For quantitative Real Time RT-PCR assays (RT-qPCR) a standard curve for ZIKV RNA copies was prepared from a previously titrated virus stock (range 10^1^ to 10^6^ PFU/mL). Plaque assays were performed on Vero E6 cells. Briefly, cells were seeded at a density of 3 × 10^5^ cells per well in standard 24 well plates and infected with serial dilutions of either cell culture supernatant from infected hiNPCs or virus stocks (for virus titration assays). After 2 h at 37°C the inoculum was removed and the cells were washed with PBS. Then the cellular monolayers were overlaid with DMEM 2% containing 1.5% CMC (carboxymethyl cellulose). Five to seven days later, the wells were washed with PBS. Afterwards, the cells were fixed with 4% formaldehyde for 10 min and stained with 0.1% crystal violet for plaque visualization.

### Statistical Analysis

The Mann-Whitney unpaired test was used to compare continuous variables. Results were expressed as Tukey box-and-whisker plots showing median, upper and lower quartile, minimum, and maximum values. Outliers are represented by dots outside the 1.5 interquartile range of the 25 respective 75 percentile. All *p*-values were two sided with a significance level of 0.05. Calculations were performed using GraphPad Prism 7 software. Cytokines and chemokines data are represented by mean ± SD. Statistical analysis was performed by unpaired Student's *t*-test using the GraphPad 7 software.

## Results

### Transcriptional Changes in ZIKV-Infected hiNPCs

**Figure 1:**
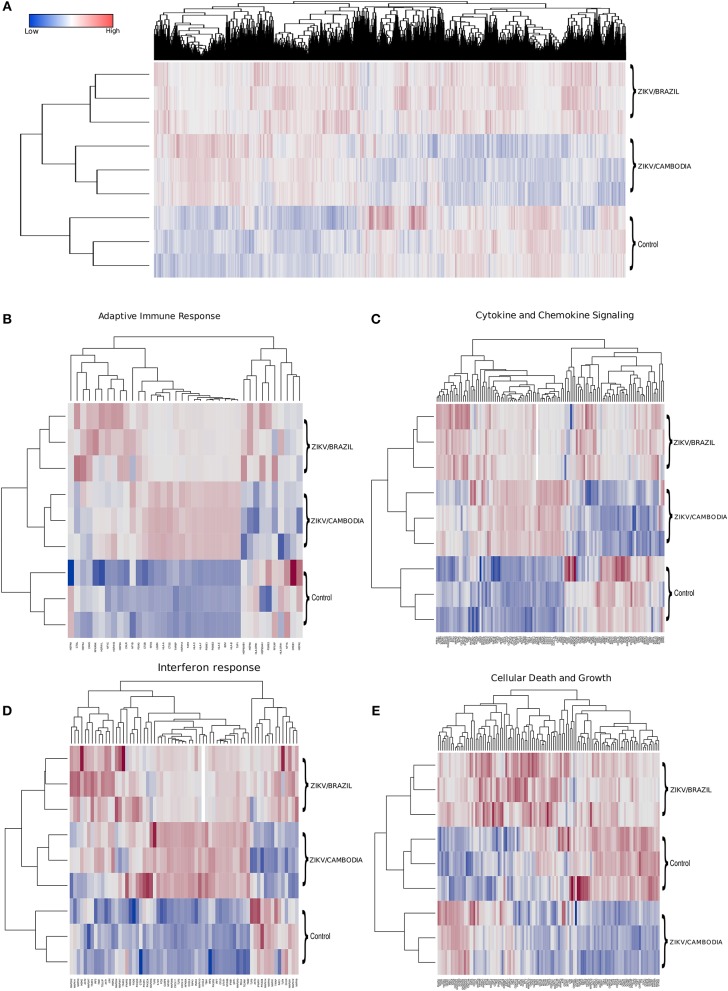
Transcriptional changes in hiNPCs induced by ZIKV infection. **(A)** Hierarchical clustering and heatmap of genes that are differentially expressed between non-infected control, ZIKV/Brazil and ZIKV/Cambodia infected hiNPCs. **(B–E)** Hierarchical clustering of heatmaps presented as normalized read-counts and represented according to different groups of KEGG analyzed pathways. **(B)** Adaptive immune response. **(C)** Cytokine and chemokine signaling. **(D)** Interferon response. **(E)** Cell death and growth. Heatmaps are representative of all identified expressed transcripts, scale bar represents gene expression of normalized reads counts for each individual triplicate. Color gradient ranges from blue (low RNA-Seq read counts) to red (high RNA-Seq read counts). Single panel lines are representative of individual infection experiments as stated for ZIKV/Brazil, ZIKV/Cambodia and control group (*n* = 3). Also see Figures S2–S4, which represent STRING functional protein networks analysis for the genes represented in the heatmaps.

To identify the transcriptional profile that may account for the differentially activated host response during infection with a contemporary vs. an ancestral ZIKV Asian strain, here denominated ZIKV/Brazil and ZIKV/Cambodia, respectively, hiNPCs were infected, and after 48 h total RNA was extracted and analyzed using the Illumina NextSeq 550 sequencing platform. Figure 1A shows the global expression changes, represented by normalized RNA-Seq read counts among all the transcripts identified as significantly changed, only if the fold change (log2) was >2 (up or down) and the corrected *P*-value was less or equal than 0.05, in comparison to the control group. Overall, of all the identified genes, both strains induced profound transcriptional modifications. Thus, to better classify those genes that may account for the different immune response induced by both strains, we categorized these identified transcripts according to KEGG pathways. The clusters of differentially expressed genes that were either up or downregulated included several key cellular processes that were related to adaptive immune response, cytokine and chemokine signaling, interferon response, and cell death and growth (Figures 1B–E). Among the modulation on genes induced by both strains, we identified the upregulation of innate immune regulatory molecules, including several C-X-C motif chemokines, *STAT* genes, *MAPK* pathway, as well as transcripts for *TLR3, IRF7*, and other type-I IFN pathway-related genes. Adaptive immune response genes were also upregulated (Figure 1B). Comparative analysis of the transcriptional signature of infected hiNPCs demonstrated that ZIKV/Cambodia infection resulted in a higher modulation of specific gene clusters, including several C-X-C motif chemokines and type-I IFN response genes (Figures 1C,D). On the other hand, ZIKV/Brazil induced a more pronounced modulation of cell death and cell growth-related genes (Figure 1E).

**Figure 2:**
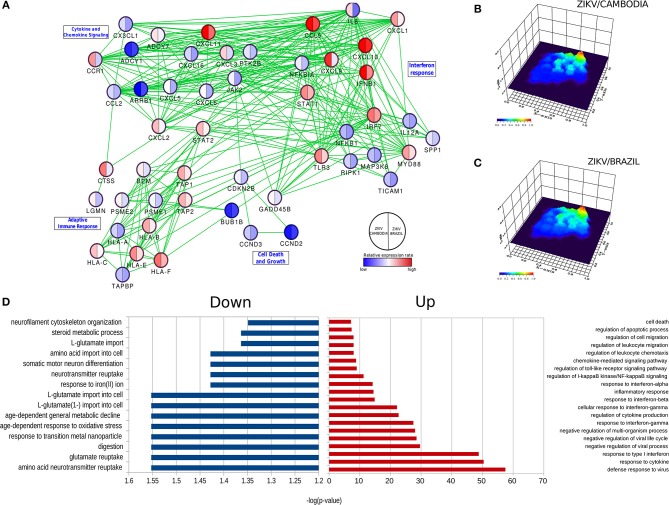
ZIKV-dependent specific cellular processes induced in hiNPCs. **(A)** Specific networks induced by ZIKV/Brazil (right) and ZIKV/Cambodia (left) infected hiNPCs. Selected modules from the protein-protein interaction network were grouped according to KEGG pathways. Color gradient ranges from blue (down-regulated in infected cells) to red (up-regulated in infected cells), and it represents the mean fold change (log10) in expression levels relative to control (non- infected cells). **(B)** Gene network (from protein-protein interaction network) represented by a three-dimensional network topology view evidencing a strong upregulation of the interferon response in ZIKV/Brazil infected hiNPCs. **(C)** Gene network (from protein-protein interaction network) represented by a three-dimensional network topology view evidencing a strong upregulation of the interferon response in ZIKV/Cambodia infected hiNPCs. Color gradient represents the transcriptional activity of infected cells from blue (low activity) to red (high activity). **(D)** Gene Ontology (GO) enrichment analysis in “Biological Process” category statistically overrepresented among all differentially expressed genes. GO terms were represented by 2-fold enrichment value, with *p*-values <0.05. Infection experiments and control groups were performed in triplicate (*n* = 3).

Predictive network analysis suggested that a significant portion of the differentially up-regulated genes comprehends a set of common pathways (Figure 2A and Figures S1–S4). Overall, the majority of genes that displayed a significant difference between control and infected cells were upregulated (Figure 2D). With regards to downregulated pathways, we observed changes mostly in genes related to metabolism and neurotransmitters (Figure 2D). Among all these processes there was a highly up-regulated notable gene cluster, the type-I IFN response. This cluster includes transcripts for *IFNB1, STAT1, IRF3, IFNAR2, IRF7*, and *TLR3* (Figures 1D, 2D). Other transcripts were also detected at increased levels, including chemokine transcripts *CXCL9, CXCL10* (among the top-ranked expressed genes), *CXCL1*, and *CCL5*. The network of highly modulated genes was further analyzed with the ViaComplex software, which compared the transcriptional profile of infected vs. uninfected cells plotting the mean expression values (Z-axis) as a 3D landscape topographical view, this analysis showed the cluster of type-I IFN response as the highest level of expression induced by infection (Figures 2B,C). In general, both strains were able to upregulate the type-I IFN response, however, we found that ZIKV/Cambodia induced a more robust modulation. Together, these results suggest that the ZIKV induced transcriptional changes are, at least in part, strain dependent.

**Figure 3:**
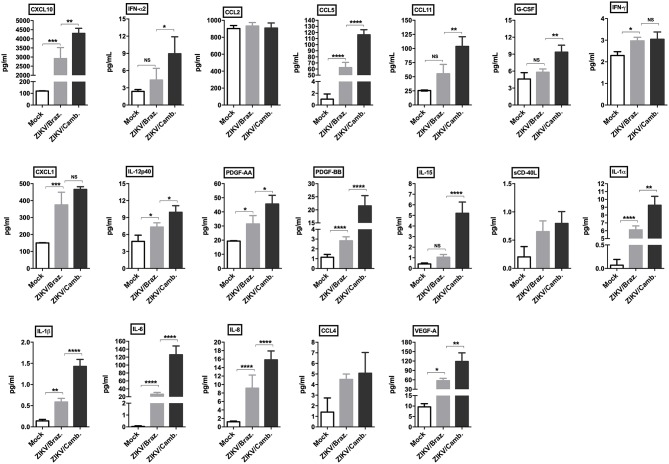
Induction of cytokine and chemokine production by ZIKV infection in hiNPCs. Individual cytokine and chemokine levels (as indicated on figures) were measured in hiNPCs cell-free supernatants from mock (non-infected), ZIKV/Brazil and ZIKV/Cambodia at 72 h post infection. Data indicate mean and SD of experiments performed in triplicate (*n* = 3), values are represented in picograms per milliliter (pg/mL). Groups were compared using non-parametric One-Way ANOVA. *P < 0.05, **p < 0.01, ***P < 0.001, ****P < 0.0001, NS, non-significant.

To confirm this inflammatory transcriptional profile we quantified the concentration of cytokines, chemokines and growth factors in hiNPCs supernatants through a multiplex assay, which included several soluble markers. We compared the levels of these markers induced after infection by both strains in a single time point (72 hpi). We observed a strong significant modulation of the pro-inflammatory cytokines IL-1α/β, IL-6, and IL-8, and the chemokines CCL5, CXCL10, and CXCL1, induced by both strains. ZIKV/Cambodia induced significantly higher levels of IFN-α2, compared to ZIKV/Brazil infected hiNPCs. Also, ZIKV/Cambodia infection was associated with significantly enhanced levels of CXCL10, CCL5, CCL11 and the pro-inflammatory cytokines IL-6, IL-8, IL-15, IL-12p40, and IL-1α/β (Figure 3). Overall, inflammatory markers assessed by this analysis correlated with the transcriptional profiles in infected hiNPCs.

**Figure 4:**
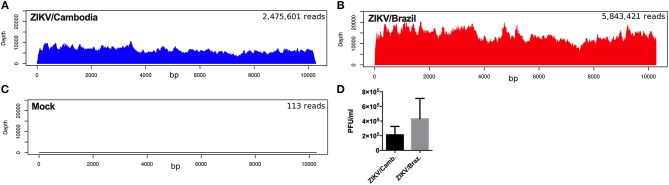
ZIKV replication in hiNPCs. Sequencing results of the infected hiNPCs were mapped to the reference genome of Zika virus (x-axis). The depth of coverage achieved from infected hiNPCs cultures and mock (non-infected control cultures) was calculated for each condition as the total number of reads (average depth), mapped against a reference genome. ZIKV/Brazil infected hiNPCs reads were mapped against the reference genome: *ZIKV/Brazil/PE243/2015 (GenBank: KX197192)*, ZIKV/Cambodia infected hiNPCs were mapped against the reference genome *ZIKV/Cambodia/FSS13025/2010 (GenBank: JN860885.11)*. **(A)** Mock–non-infected cells. **(B)** hiNPCs infected with ZIKV/Brazil, analyzed at 48 h post infection. **(C)** hiNPCs infected with ZIKV/Cambodia, analyzed at 48 h post infection. **(D)** hiNPCs were infected in triplicate (*n* = 3) with either ZIKV/Brazil or ZIKV/Cambodia at a multiplicity of infection of 0.1. Infectious virus recovery in supernatant at 48 h post infection was determined by plaque titration assay. Values are expressed as mean of plaque forming units per milliliter (PFU/mL). Bars are representative of individual infection experiments performed in triplicate (*n* = 3).

To further explore if the enhanced inflammatory activity from ZIKV/Cambodia infected cells was associated with a difference in virus replication, we quantified the average reads depth of the ZIKV genome from infected and non-infected hiNPCs. As expected, no ZIKV genome reads were observed in the control group (Figure 4A). However, nearly complete ZIKV genomes were recovered from infected hiNPCS, with a high coverage from both strains (coverage above 99.9%). NGS results demonstrated that ZIKV/Cambodia gave a much lower read counts average (depth below 10,000) compared to ZIKV/Brazil infected cells (depth above 10,000) (Figures 4B,C). Albeit lower, ZIKV/Cambodia sequencing depth RNA levels, were not correlated to a significant increased viral load (infectious particles) in infected hiNPCs supernatants, analyzed at 48 hpi (Figure 4D). Thus, despite the minor differences in virus replication, it is suggestive that the magnitude of the inflammatory response may interfere with the viral titers, though key pathways are activated regardless of the virus strain.

### ZIKV Infection of Human Neuroblastoma Cells Also Induce Chemokines Release

**Figure 5:**
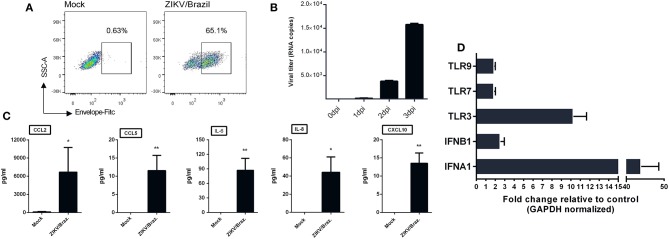
ZIKV infection of human neuroblastoma cells results in cytokine release. **(A)** SH-SY5Y cells were infected with ZIKV/Brazil (MOI = 1), harvested at 2 days post-infection (dpi), stained with anti-flavivirus envelope (E) protein 4G2 and anti-mouse conjugated with FITC, and analyzed by flow cytometry (FACS). **(B)** SH-SY5Y cells were infected with ZIKV/Brazil (MOI = 1) and the supernatants were processed for viral RNA extraction and qRT-PCR at different days post infection. **(C)** SH-SY5Y cells were infected with ZIKV/Brazil (MOI = 0.5), supernatants were harvested at 3 dpi and cytokines/chemokines production levels were quantified by Cytometric Bead Array. **(D)** RT-qPCR results of selected genes related to type-I IFN response. Relative expression (fold change) of mRNA transcripts are shown from infected SH-SY5Y cells (analyzed at 48 h after infection) relative to control group. Mock—cell not infected. Data are represented by mean ± SD. Figures are representative of three independent experiments, performed in triplicate (*n* = 3). *p < 0.05, **p < 0.01, by unpaired Student's *t*-test.

Given the ability of ZIKV infection to up-regulate chemokines and cytokines transcripts in hiNPCs, we determined the susceptibility of a distinct neuronal cell line. For this, we opted to evaluate the feasibility of human neuroblastoma cells (SH-SY5Y cells) to respond to ZIKV infection by increasing cytokines/chemokines production. First, to confirm infection, undifferentiated SH-SY5Y cells were infected and analyzed by FACS at 2 dpi, positive viral envelope protein (E) staining was observed in a large percentage (65.1%) of the infected cells (Figure 5A) and viral RNA, detected in the supernatant, increased over time (Figure 5B), confirming that these cells were susceptible to infection. Next, we evaluated the presence of a set of cytokines and chemokines in the supernatants using two different sets of human CBA assays. The results show increased production of IL-8, CXCL10, CCL2, CCL5, and IL-6 (Figure 5C) at 3 dpi, indicating the ability to induce an active pro-inflammatory response. No other cytokines were detected at significantly increased levels (data not shown). Infection also results in enhanced expression of several IFN stimulated genes, especially *IFNA1, IFNB1*, and *TLR3* transcripts (Figure 5D).

### CNS Inflammatory Profile of ZIKV Confirmed Microcephaly Cases

The increased type-I IFN and chemokine responses observed from ZIKV infection are common features of viral infections including ZIKV, however, in the CNS, a hyperactivation or a sustained inflammatory process may result in extensive tissue damage, especially in the context of a developing brain. Thus, to better characterize the ZIKV inflammatory profile we assessed the CNS immune profile of confirmed ZIKV-induced microcephaly and severe microcephaly human cases (here, collectively denominated “microcephaly”). From a total 2,334 CZS suspected cases from September 2015 to May 2016 in Northeastern Brazil, we were able to identify 27 healthy control patients (no brain alterations) and 51 cases of microcephaly, based on clinical diagnosis, with CSF sample availability (characterized by a previous collected sample stored at the laboratory). Among the microcephaly cases included in our study, 40 (78.4%) were diagnosed as severe microcephaly (head circumference <3 SD on the Fenton growth chart) and 11 as microcephaly (head circumference <2 SD). Complete information and patient characteristics and laboratory results are summarized in Table 1. Additional laboratory data are summarized in Table S1 and Supplementary Material. ZIKV infection was diagnosed by the presence of anti-ZIKV IgM in the CSF in all of the 51 confirmed microcephaly cases. Among these 51 cases, the presence of ZIKV RNA (rRT-PCR) was identified in only two samples (Table S1). Importantly, no positive anti-ZIKV IgM or rRT-PCR CSF samples were observed in the healthy control group. STORCH testing for common congenital infections was performed for all subjects, and no IgM reactive samples were documented (Table S1 and Supplementary Material). Based on this, other congenital common infections were ruled out, and the final diagnosis was microcephaly associated with maternal ZIKV infection.

**Table 1:** Characteristics of patients included in the study.

	**Healthy control**	**Microcephaly**
**Neonates, total number of subjects (%)**	**27 subjects**	**51 patients**
Male	10 (37)	22 (43)
Female	17 (63)	29 (57)
**Gestational age at birth, (%)**		
Pre-term	5 (18.5)	5 (9.8)
Term	21 (77.8)	44 (86.2)
Post-term	1 (3.7)	2 (4)
**Birth weight, grams (%)**		
>2,500 g	19 (70.4)	39 (76.5)
>1,500–2,499.9 g	8 (29.6)	10 (19.6)
<1,500 g	0	1 (1.95)
Not informed	0	1 (1.95)
**Head circumference (%)**		
Normal	27 (100)	0
<2 SD (microcephaly)	0	11 (21.6)
<3 SD (severe microcephaly)	0	40 (78.4)
**Mothers (total number of subjects)**	**27**	**51**
**Reported rash during pregnancy (%)**		
No rash	26 (96.3)	35 (68.6)
First trimester	0	9 (17.6)
Second trimester	1 (3.7)	1 (1.95)
Third trimester	0	3 (5.9)
Yes, unknown period	0	3 (5.9)

*A total of 27 neonates with a discarded diagnosis of microcephaly (healthy controls) were included in this study. Among the confirmed microcephaly and severe microcephaly cases, a total of 51 neonates, which had CSF samples available, were included for further cytokine investigation. All of the neonates had CSF samples collected no later than 4 weeks after birth*.

**Figure 6:**
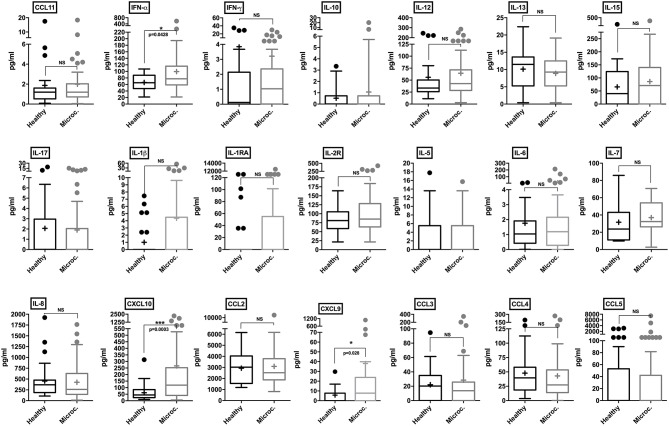
Cytokine and chemokine profiles in the CSF of control and microcephaly cases. Individual cytokine and chemokine levels (as indicated on figures) were measured in the CSF of control (*n* = 27) and ZIKV-induced microcephaly cases (*n* = 51), and are represented by Tukey box-and-whisker plots showing median, upper and lower quartile, minimum, and maximum values (picograms per milliliter-pg/mL). Outliers are represented by dots outside the 1.5 interquartile range of the 25 respective 75 percentile. Mean values are indicated by a plus (+) sign. Mann-Whitney unpaired test *P < 0.05, ***P < 0.001, vs. healthy control (exact *p*-values are described in figures). NS, non-significant vs. healthy control.

CSF samples from healthy control subjects and microcephaly neonate cases were analyzed employing a panel of 25 different human cytokines and chemokines. Through our analysis, microcephaly-associated CSF samples showed significantly higher levels of IFN-α (*p* = 0.0428), compared to the control group. Albeit slightly higher, there was no significant difference in the levels of IL-1β and IL-1RA (*p* = 0.054 and *p* = 0.10, respectively). The pro-inflammatory cytokines IFN-γ, IL-6, and IL-8 were similar between the groups. Among chemokines, we did not observe a significant difference in the levels of CCL2, CCL3, CCL4, CCL5, and CCL11. Interestingly, consistent with the *in vitro* results, two CXC chemokine ligands were found at significantly higher levels in the CSF samples from microcephaly cases: CXCL9 and CXCL10 (*p* = 0.028, and *p* = 0.0003, respectively). Among T-cell activation related cytokines, we did not observe a cytokine signature associated with a predominant helper T-cell response. We also observed no difference in IL-5, IL-10, IL-13, IL-15, IL-17, and interleukin-2 receptor (IL-2R) production between microcephaly cases compared to the control group (Figure 6). Four cytokines (GM-CSF, TNF-α, IL-2, and IL-4) were below the detection limit (data not shown). Altogether, these observations show that the transcriptional changes induced by ZIKV infection results in respective protein levels increase *in vitro* and that this inflammatory signature is also present in the CNS of ZIKV-induced microcephaly cases. Moreover, our data suggest that children suffering from ZIKV-induced microcephaly may experience long-term or chronic neuroinflammation.

## Discussion

Currently, the underlying pathophysiological mechanisms of ZIKV-induced microcephaly and CZS remain poorly understood. It has been documented that ZIKV infection leads to a reduction in NPCs numbers as a result of apoptosis (29), cell cycle arrest (30), and premature differentiation (31), consequently impairing neuronal development. Although NPCs are the main targets of ZIKV replication effects, infection in isolated cell models are frequently underexplored regarding inflammatory responses (32). Here, we employed different approaches to better characterize the CNS inflammatory response induced by ZIKV infection. First, through the use of hiNPCs, we observed a strain-dependent modulation of host genes, including the induction of several ISGs. Next, we confirmed that experimental infection of hiNPCs leads to extensive chemokine and cytokine release, consistent with the induced transcriptional profile. Since the manipulation of stem cells may result in differentiation variability between distinct cell lines, laboratories and in variations in differentiation efficiency (33), we opted to include a second cell line to model ZIKV infection. Human neuroblastoma cells have the advantage of being inexpensive, consistent and reproducible neuronal cell model (34). These cells supported a productive ZIKV replication cycle, with a similar inflammatory profile. However, an important finding from our work is that the *ex vivo* profiling of the CNS inflammation in ZIKV-induced microcephaly infants supports our *in vitro* experimental data.

As reported by others, the inflammatory response elicited by ZIKV is strain-dependent, where African induces a much higher inflammatory response than Asian strains (35). Additionally, African strains are less effective in inhibiting type-I IFN responses (36). In our model, we found a specific signature induced by ZIKV/Brazil, a previously characterized South American strain (26). Importantly, this virus was isolated from a highly endemic area (northeastern Brazil), concomitantly at the peak of microcephaly cases in 2015. Thus, we can assume that ZIKV/Brazil represents a virus strain-specificity (same strain circulating during the initial microcephaly outbreak). Evidently, infection with both strains led to an inflammatory immune response in hiNPCs, however differences in the magnitude of inflammatory cytokines were found to be strain-specific, which may help to explain why a higher incidence of microcephaly was first observed in northeastern Brazil. A reasonable explanation is that the lower magnitude of inflammation induced by the innate immune responses may inefficiently limit viral replication, since the reduced type-I IFN response may result in augmented higher viral burden or chronification of the infection. Interestingly, as reported by others, ZIKV Brazilian strains share characteristics of viruses that do not induce a robust innate immune activation (37), which may render these strains more effective at establishing a persistent infection or increased capacity to cross the placenta and/or to invade the CNS. Here, although ZIKV RNA levels were significantly lower in ZIKV/Cambodia compared to ZIKV/Brazil infected hiNPCs, we observed only a slight difference in the presence of infectious virus at 48 hpi, and thus a complete virus growth curve would be more enlightening. Therefore, our results contribute to a better understanding of ZIKV immunopathological processes, and they are in agreement with a previous report where an American strain of ZIKV results in an enhanced viral load and a more severe microcephaly phenotype in mice (38). Importantly, as demonstrated in an independent study, a distinct ZIKV Brazilian strain showed a delayed production of infectious virus *in vitro*. By comparing ZIKV/Cambodia to ZIKV Brazil Fortaleza (*GenBank: KX811222*) the authors found that ZIKV/Brazil reached a virus production peak at a later time point (72 h), whereas in early times this difference was not so evident (37).

Through the transcriptional profile analysis and multiplex immunoassays, we identified key major cellular signaling pathways and inflammatory markers. Transcriptomic data of experimentally ZIKV infected cells have been generated by several independent groups (39–41), however, given the large variability of the cell lines employed, time of infection, virus strains and amount of infectious particles on each approach, combinatory analysis of such data becomes challenging. We demonstrated that IFN-α, CXCL10, and CXCL9 levels were significantly higher in CSF samples obtained from ZIKV-induced microcephaly cases, compared to healthy control subjects. It is important to note that all CSF sampling was performed right after birth, up to a maximum of 4 weeks of life, ensuring that the presence of soluble immune mediators is representative of a sustained inflammatory process. High variation in expression levels of a few cytokines (notably IL-1β and IL-1RA) was observed, potentially demonstrating that we missed the detection of specific perturbations as the result of a single time point sampling (after birth). Also, several cytokines were below the detection limit, indicating their low levels of expression in the CSF. We could not predict exactly when the infection occurred, which may explain some of the variations observed. However, even though the virus was not detected in most of the subjects analyzed, as illustrated by the large proportion of rRT-PCR negative CSF samples, we can assume that the resulting inflammation is persistent.

Remarkably, several different congenital infections lead to a common clinical presentation, collectively denominated TORCH (Toxoplasmosis, Other, Rubella, Cytomegalovirus, and Herpes) these congenital infections also result in microcephaly and cerebral calcifications. Overall, these pathogens are highly neurotropic, but first, they must cross the placenta to induce fetal damage (42). Currently, it remains unclear how ZIKV can reach the fetal compartment, as well as the exact fetal tissues targeted. Albeit ZIKV antigens and viral RNA were detected almost exclusively in the brains of infants and fetuses with microcephaly (7, 8, 43) no descriptions regarding the neural tissue inflammation has been reported. Interestingly, Aicardi-Goutières syndrome (AGS) resembles the clinical findings of ZIKV-induced microcephaly. AGS patients present brain calcifications, changes in white matter, cerebral atrophy and the laboratory findings include increased levels of IFN-α in the CSF (44). Mechanistically, AGS patients have been classified in the group of type-I interferonopathies, which comprise a group of genetic (Mendelian) disorders caused by a sustained type-I IFN response during the embryonic development. Experimentally, the chronic exposure of astrocytes to high levels of IFN-α resulted in reduced cell proliferation, increase in antigen-presenting genes and down-regulation of pro-angiogenic factors (45). Besides, the transgenic expression of IFN-α in the CNS of mice induces inflammation and neurodegeneration, similar to that seen in AGS (46–48). Based on this we can assume that the sustained levels of type-I IFN, here described, may play a role in ZIKV-induced microcephaly contributing to the induction of detrimental developmental effects.

Recently published reports from animal models, which explored the effects of ZIKV during pregnancy, required the blockade of type-I IFNs to achieve susceptible hosts (19, 49). Thus, the absence of type-I IFN signaling in ZIKV animal experiments lacks an important component in studies related to ZIKV immunopathogenesis. In agreement with our data, the induction of type-I IFN signaling, as a result of ZIKV infection in mice, led to increased apoptosis in the placental labyrinth, abnormal maternal-fetal barrier, and fetal hypoxia. Moreover, type-I IFN treatment of human midgestation villous explants led to abnormal villous structures, which are strongly associated with growth restriction and spontaneous abortions (50). Albeit of great relevance, the authors explored the effects of type-I IFNs exclusively on the developing placenta. Here, we propose that type-I IFNs may play an important role in developmental defects caused by ZIKV, specifically in the context of brain damage, as a result of chronic local inflammation.

As reported, ZIKV non-structural proteins are effective in inhibiting type-I IFN, which favors virus replication and may lead to viral persistence in different body compartments (51–53). In fact, recent reports describe ZIKV persistence in infants (54, 55) and adults (56). Here, we hypothesize that peripheral type-I IFN inhibition is an essential step that facilitates CNS invasion. Once in the CNS, the virus induces a sustained, suboptimal type-I IFN response that leads to extensive neuroinflammation and tissue injury. Interestingly, activation of type-I IFN signaling in brain endothelial and epithelial barriers results in CXCL10 release into the brain parenchyma and this signaling cascade was recently correlated to “sickness behavior,” a common set of symptoms due to viral infections (57). Of note, CXCL10 and CXCL9 can be induced by both, type-II (IFN-γ) and type-I IFNs (58). Increased levels of IFN-α and CXCL10 in the CSF of AGS patients were already documented, and these findings were associated with the absence of IFN-γ (59). Mechanistically, CXCL9 and CXCL10 bind to CXCR3, a receptor primarily expressed on T cells and NK cells (60) also found in NPCs (61). Among other effects, CXCL10 treated neurons developed increased membrane permeability, which was followed by caspase-3 dependent apoptosis (62). In fact, ZIKV infection leads to extensive caspase-3 activation in different animal and *in vitro* models (19, 29, 63, 64). This correlation becomes more apparent considering that IFN-α/β treatment leads to enhanced calcification of cultured human vascular smooth muscle cells (65), indicating that IFN-α promotes the generation of calcium deposits and supports the proposition that IFN-α acts directly on ZIKV-induced microcephaly calcifications, a frequently reported clinical finding. Thus, we suggest that in the CNS, induction of IFN-α by ZIKV infection acts to further up-regulate the local chemokine response and consequently, apoptosis. On the other hand, direct IFN-independent induction of CXCL10 has been documented for different RNA viruses (66–68). Interestingly, CXCL10 elicits apoptosis in fetal neurons, dependent on intracellular Ca(2+) increase and caspase-3 activation (69). Based on this, we propose a model in which these inflammatory mediators act synergistically contributing to most of the alterations observed in microcephaly cases. To our knowledge, we are the first to demonstrate a more detailed neuroinflammation profile from human cases of ZIKV-induced microcephaly. Collectively, our data corroborate other independent findings that implicate type-I IFNs as a potential modulating factor in ZIKV-associated pregnancy complications.

## Data Availability

RNA-Seq data are available at NCBI BioProject under accession number PRJNA551246. Data supporting this study can be found at Supplementary Files. A complete description of methods can be requested from the corresponding author, further information supporting this study can also be made available.

## Author Contributions

ML and RC performed the sample processing, Luminex assays, and analysis. ML, EA, RA, PF, PC, and CA-S contributed to the sample classification and laboratory diagnosis. LM performed the SH-SY5Y cells infection experiments, FACS, qRT-PCR analysis, and Luminex assays. MD, LD'A, KC, and JW performed the hiNPCs infection experiments and RNA-Seq assay. AR performed the RNA-Seq and transcriptomics analysis. PC performed the virus quantification assay. AL-A, MG, and RF performed the statistical analysis of cytokines and chemokines profile in human samples. EM, AL-A, RF, CD, AK, VN, and MB-N contributed to the data interpretation and discussion. RF conceived the study, supervised the study, and wrote the manuscript. All authors discussed the results and contributed to the final manuscript.

### Conflict of Interest Statement

The authors declare that the research was conducted in the absence of any commercial or financial relationships that could be construed as a potential conflict of interest.
