# Supplementary material for: The Transcriptional and Protein Profile From Human Infected Neuroprogenitor Cells Is Strongly Correlated to Zika Virus Microcephaly Cytokines Phenotype Evidencing a Persistent Inflammation in the CNS
Source: Front Immunol. 2019 Aug 16;10:1928. doi: 10.3389/fimmu.2019.01928 (PMC6707094; doi:10.3389/fimmu.2019.01928)
Supplement: Supplementary file 1 [file Data_Sheet_1.pdf]

The transcriptional and protein profile from human infected neuroprogenitor cells is strongly correlated to Zika virus microcephaly cytokines phenotype evidencing a persistent inflammation in the CNS

Morganna C. Lima[1#], Leila R. de Mendonça[1#], Antonio M. Rezende[1#], Raquel M. Carrera[2], Conceição E. Aníbal-Silva[1], Matthew Demers[3], Leonardo D'Aiuto[3], Joel Wood[3], Michael Griffiths[2], Antonio R. Lucena-Araujo[4], Manoel Barral-Netto[5], Elisa A. N. Azevedo[1], Renan W. Alves[1], Pablo C. S. Farias[1], Ernesto T. A. Marques[1 6], Priscila M. S. Castanha[6], Claire L. Donald[7], Alain Kohl[7], Vishwajit L. Nimgaonkar[3 8] and Rafael F. O. Franca[1*]

# these authors contributed equally to this work

1. Oswaldo Cruz Foundation/Fiocruz, Institute Aggeu Magalhães, Recife, Brazil 50.740-465.
2. University of Liverpool, Institute of Infection and Global Health, Liverpool, United Kingdom L69 7ZX.
3. Department of Psychiatry, University of Pittsburgh School of Medicine, Pittsburgh, USA 15260
4. Federal University of Pernambuco/UFPE, Recife, Brazil 50.740-465.
5. Oswaldo Cruz Foundation/Fiocruz, Institute Gonçalo Moniz, Salvador, Brazil 40296-710.
6. University of Pittsburgh, Center for Vaccine Research, Pittsburgh, USA 15260.
7. MRC-University of Glasgow Centre for Virus Research, Glasgow, Scotland, United Kingdom G61 1QH.
8. Department of Human Genetics, Graduate School of Public Health, University of Pittsburgh, USA 15260.

*Corresponding Author

Address for correspondence: Rafael F. O. Franca, Oswaldo Cruz Foundation - Fiocruz, Department of Virology and Experimental Therapy, Av. Professor Moraes Rego, s/n, Cidade Universitaria Recife, Pernambuco 50740-465, Brazil;

email: rafael.franca@cpqam.fiocruz.br

Supplementary Results

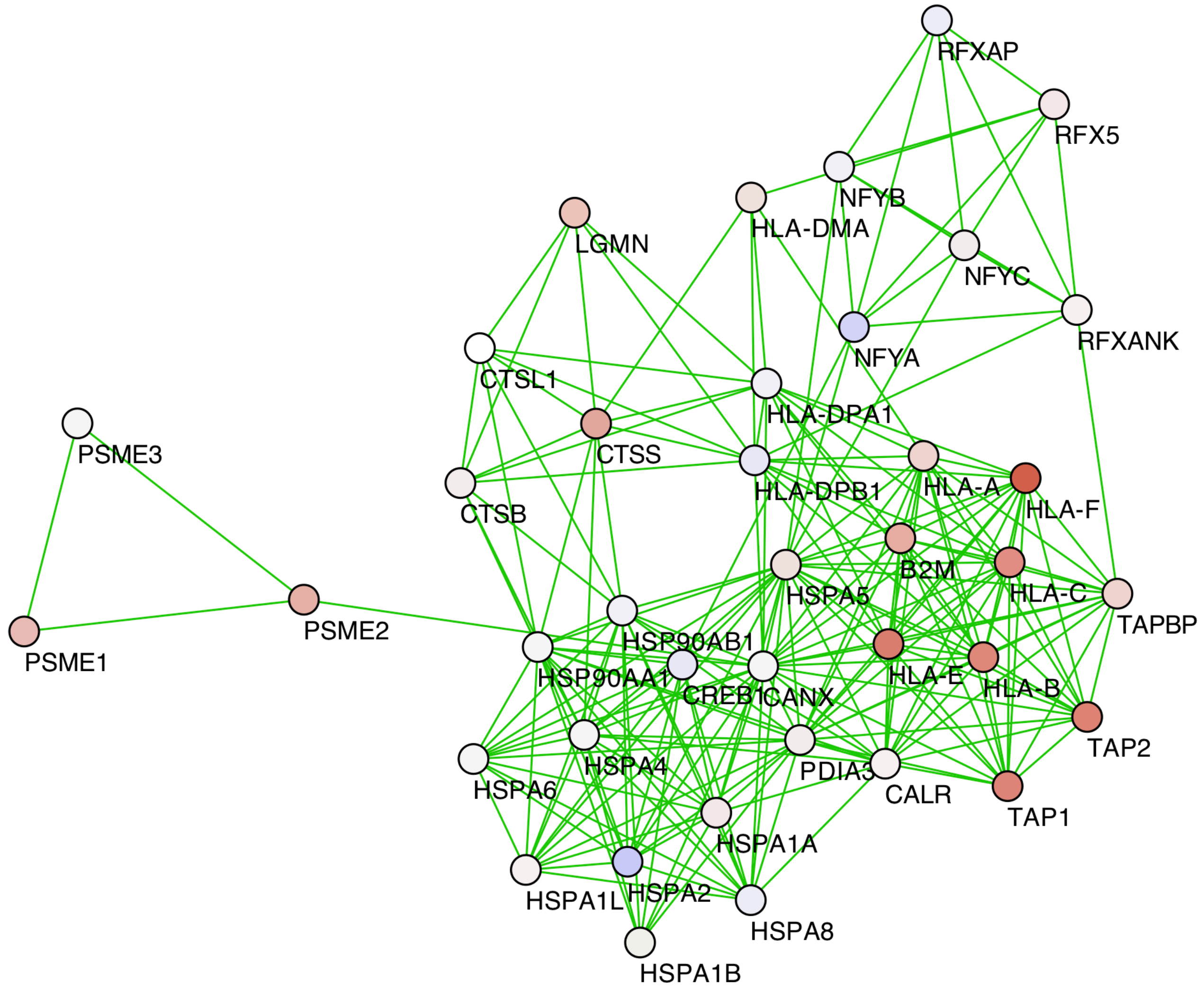

Figure S1. Protein interactions extracted from the STRING database. The minimum interaction score was equal to 0.4 for genes encoding proteins grouped into the Adaptative Immune Response category, which was created based on KEGG pathway analysis.

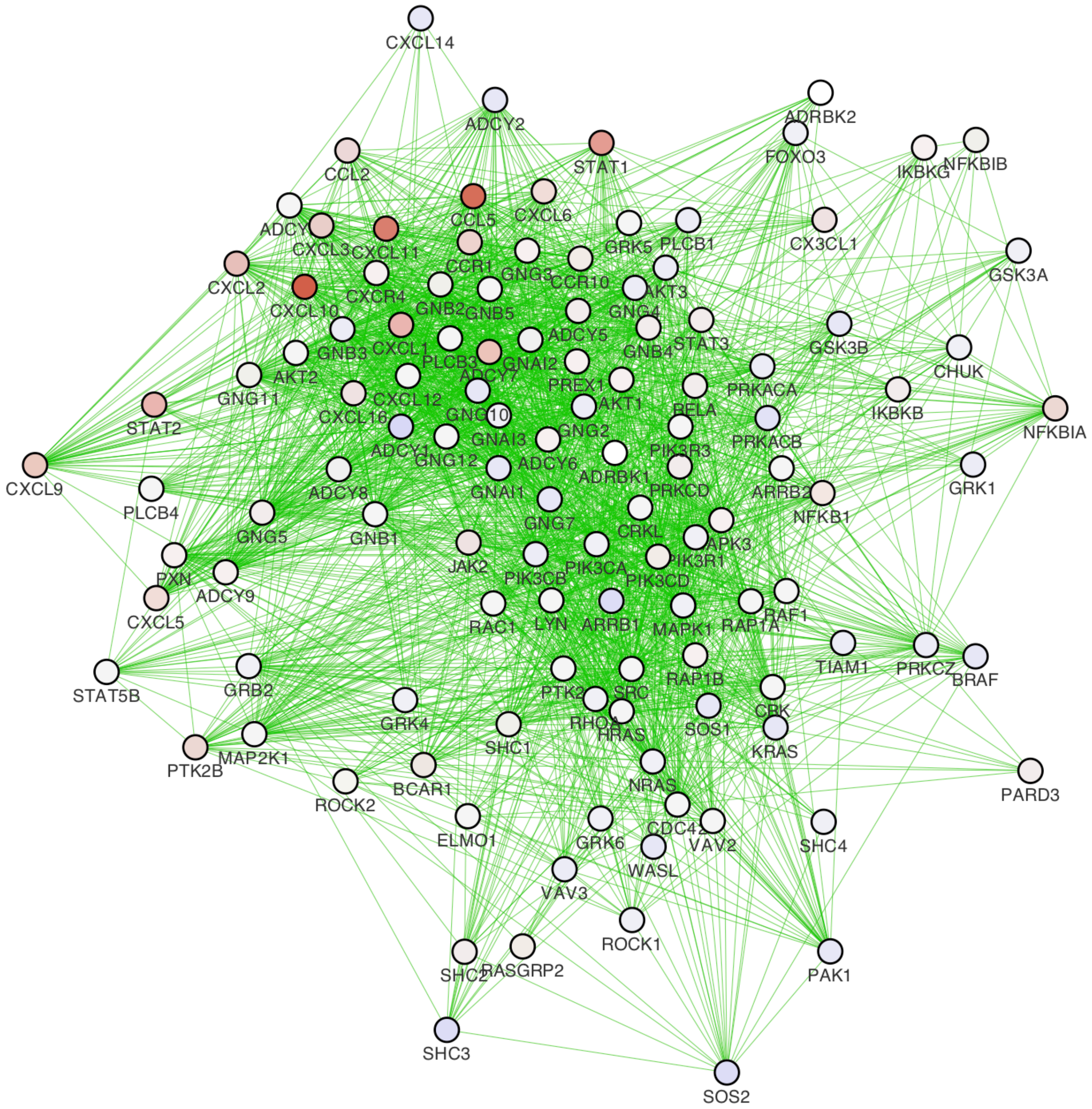

Figure S2. Protein interactions extracted from the STRING database. The minimum interaction score was equal to 0.4 for genes encoding proteins grouped into the Cytokine and Chemokine Signaling category, which was created based on KEGG pathway analysis.

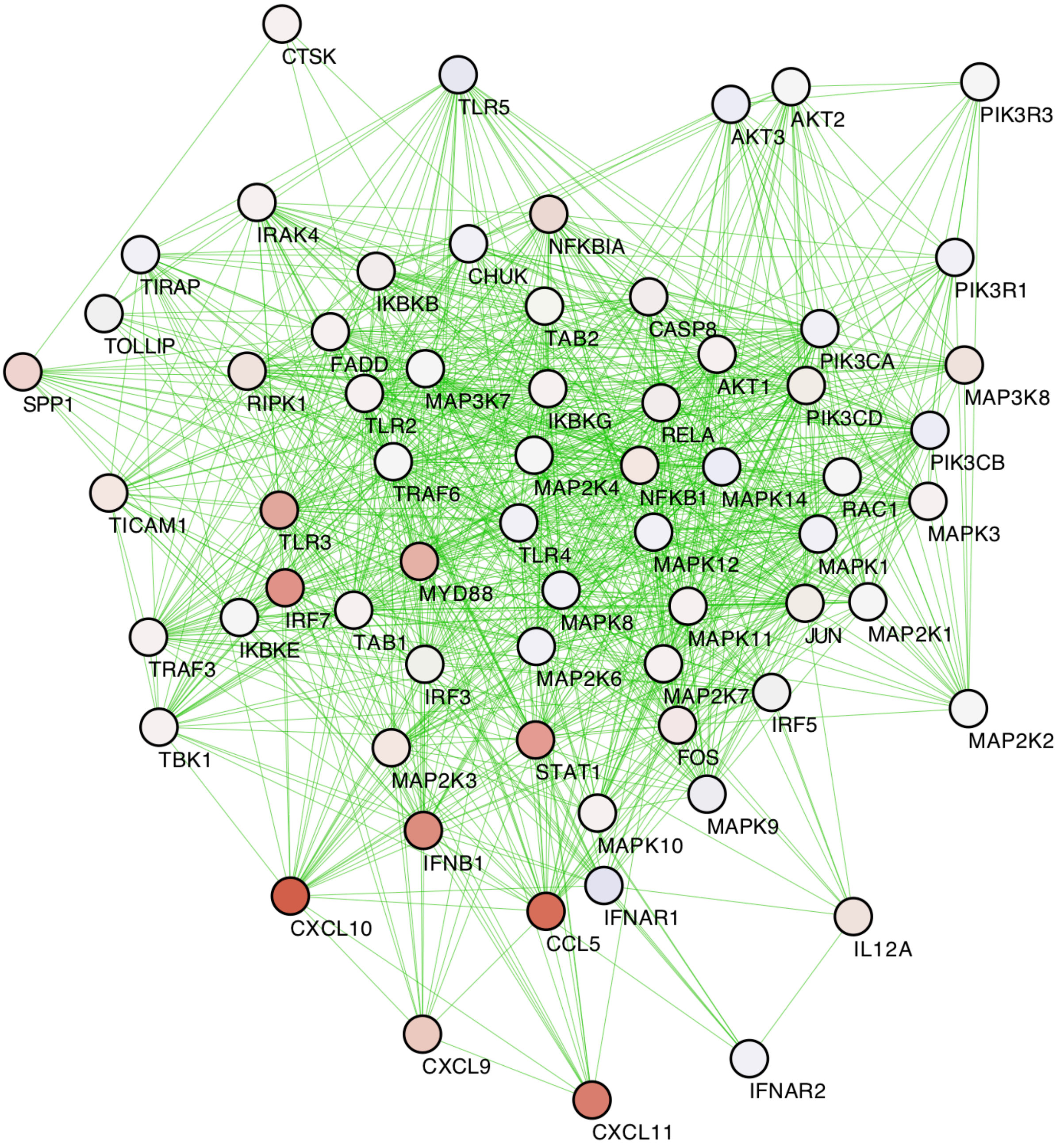

Figure S3. Protein interactions extracted from the STRING database. The minimum interaction score was equal to 0.4 for genes encoding proteins grouped into the Interferon Response category, which was created based on KEGG pathway analysis.

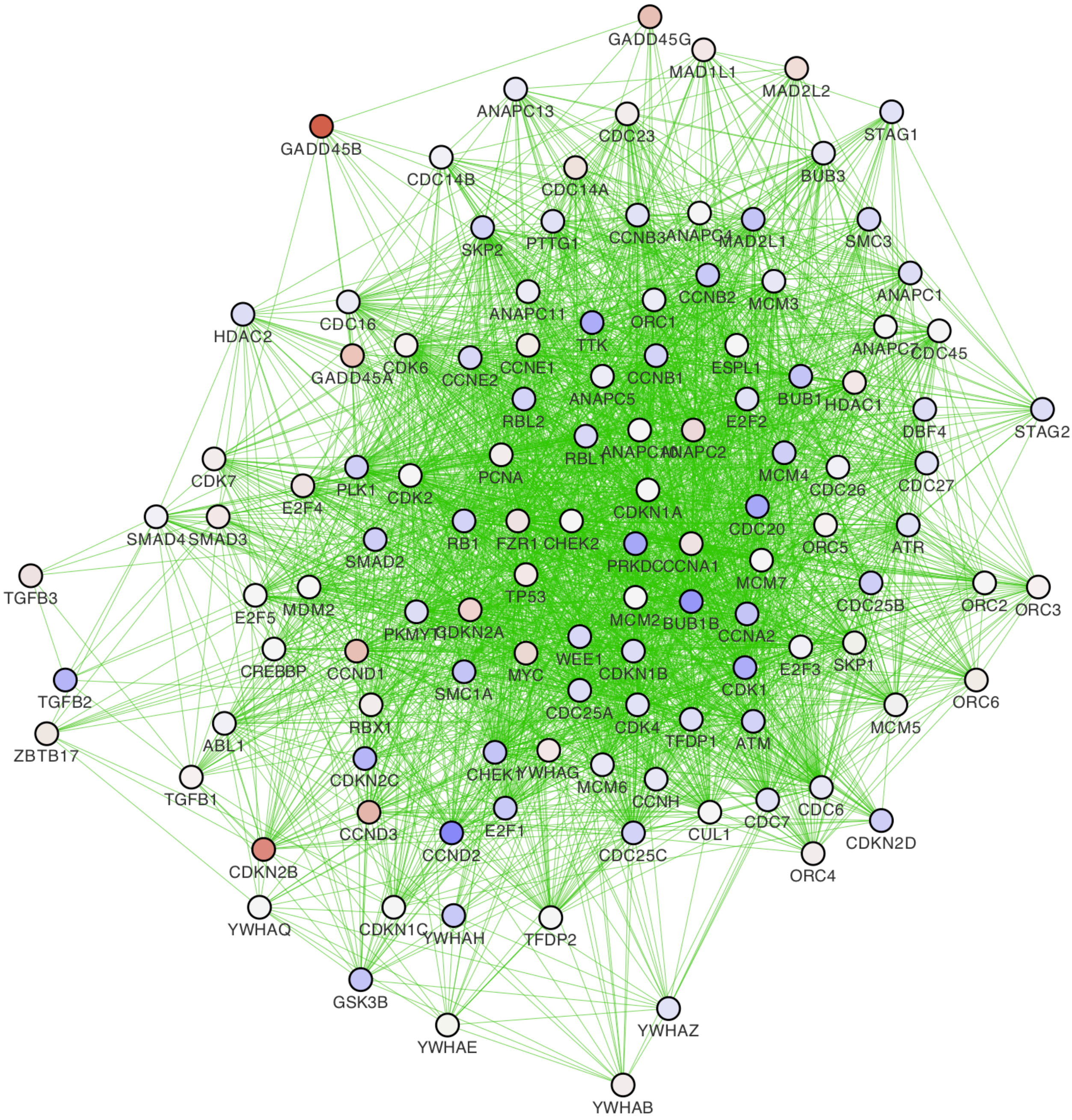

Figure S4. Protein interactions extracted from the STRING database. The minimum interaction score was equal to 0.4 for genes encoding proteins grouped into the Cellular Death and Growth category, which was created based on KEGG pathway analysis.

Healthy Controls			
	**ZIKV rRT-PCR**	**ZIKV IgM**	**STORCH**
**CSF**	0	0	Not performed
**Serum**	0	0	Non-reactive* 27 (100)
**Congenital Zika Syndrome cases**			
	**ZIKV rRT-PCR**	**ZIKV IgM**	**STORCH**
**CSF (%)**	0	51 (100)	-
**Serum (%)**	2 (3.9)	Not performed	Non-reactive* 51 (100)

Table S1. Laboratory confirmation of ZIKV and other congenital infections. STORCH (Syphilis, Toxoplasma gondii, rubella, cytomegalovirus and herpes Simplex) tests were performed using serum samples only. rRT-PCR - Real Time RT-PCR. Non-reactive - IgM negative for any tested pathogen. Complete description of laboratory results is described in Supplementary File 1.

Analyte	Detection range (lower and upper Values – pg/ml)
CCL11 (EOTAXIN)	1.42 – 3148.91
GM-CSF	2.10 – 4998.17
IFN-α	13.37 – 7966.26
IFN-γ	1.03 – 5199.82
IL-10	0.58 – 3949.96
IL-12	14.51 – 7549.90
IL-13	5.48 – 11521.22
IL-15	8.15 – 40861
IL-17	5.53 – 19624.21
IL-1β	12.6 – 7149.94
IL-1RA	18.73 – 10932.85
IL-2	3.63 – 9702.54
IL-2R	8.93 – 21249.79
IL-4	10.01 – 25191.07
IL-5	14.67 – 7998.5
IL-6	2.16 – 5744.34
IL-7	20.99 – 9892.6
IL-8	1.97 – 8897.11
CXCL10 (IP-10)	2.18 – 2860.96
CCL2 (MCP-1)	10.11 – 16000
CCL3 (MIP-1α)	9.73 – 6127.43
CCL4 (MIP-1β)	43.65 – 2600.19
CCL5 (RANTES)	2.04 – 4578.95
TNF-α	1.06 – 2492.63
CXCL9 (MIG)	20.61 – 3849.71

Table S2. Cytokines and chemokines upper and lower detection limits analyzed by Luminex using the Cytokine Human Magnetic 25-Plex Panel. Values are expressed in picograms per milliliter (pg/ml). From all CSF samples, individual cytokines were assayed in a Luminex Platform employing a commercially available kit: Cytokine Human Magnetic 25-Plex Panel (Thermo Fisher Scientific - Munich, Germany) specifically designed for quantifying human cytokines and chemokines, following the manufacturer's instructions.

Analyte	Detection range (lower and upper values – pg/ml)
EGF	3.35 – 8876.63
FGF-2	22.28 – 10275.30
CCL11 (EOTAXIN)	0.64 – 7137.23
TGF-a	0.64 – 10000.00
G-CSF	0.20 – 10209.22
Flt-3L	0.65 – 8116.86
GM-CSF	0.64 – 7404.83
Fractalkine	3.20 – 10000.00
IFN-α2	0.64 – 10000.00
IFN-γ	0.64 – 10000.00
CXCL-1 (GRO alpha)	16.15 – 17470.74
IL-10	0.64 – 7539.68
MCP-3	3.26 – 9875.63
IL12B (IL12P40)	3.20 – 10000.00
MDC	16.00 – 10000.00
IL12A (IL12P70)	0.64 – 10000.00
PDGF-AA	0.64 – 2155.19
IL-13	0.64 – 9654.14
PDGF-BB	0.85 – 9901.03
IL-15	0.64 – 10000.00
sCD40L (CD154)	0.60 – 11291.68
IL-17A	0.63 – 9931.25
IL-1RA	0.64 – 9931.25
IL-1α	0.75 – 9771.13
IL-9	0.65 – 6433.02
IL-1β	0.65 – 9559.20
IL-2	0.62 – 9838.04
IL-3	0.64 – 10000.00
IL-4	0.64 – 10000.00
IL-5	0.63 – 3613.63
IL-6	0.59 – 2016.39
IL-7	0.63 – 1582.92
IL-8	0.65 - 9494,28
CXCL-10 (IP-10)	4.09 – 9300.64
CCL2 (MCP-1)	0.65 – 3992.65
CCL3 (MIP-1α)	4.14 – 1728.73
CCL4 (MIP-1β)	0.64 – 8132.61
CCL5 (RANTES)	0.06 – 9998.40
TNF-α	0.62 – 5094.92
TNF-β	0.66 – 9702.49
VEGF-A	0.94 – 1996.72

IL-18	0.64 – 10000.00

Table S3. Cytokines and chemokines quantified in supernatant samples from mock- and ZIKV-infected hiNPCs, analyzed by Human Cytokine array / Chemokine Array 42-Plex with Luminex® xMAP® technology. Samples were harvested at 48 hpi and quantification assay was performed by Eve Technologies Corporation (Calgary, AB, Canada). Lower and upper limits of detection values are shown in picograms per milliliter (pg/ml).
